# A Novel Approach to Care Redesign Collaboration Between Emergency and Specialty Departments: Qualitative Experience Report

**DOI:** 10.2196/22028

**Published:** 2025-08-26

**Authors:** Sonia Rose Harris, Christian Rose, Samantha MR Kling, Brett A Cohen, Olga Goldberg, Hannah Walton, Darlene Veruttipong, Mohamed Alhadha, Sheneé K Laurence, Shashank Ravi, Carl A Gold, Jonathan G Shaw, Laurice Yang, Cati G Brown-Johnson

**Affiliations:** 1 Evaluation Sciences Unit, Division of Primary Care and Population Health, Department of Medicine, School of Medicine, Stanford University, 3180 Porter Drive, Palo Alto, CA, 94304, United States, 1 5155209181; 2 Department of Emergency Medicine, School of Medicine, Stanford University, Stanford, CA, United States; 3 Department of Emergency Medicine, Vanderbilt University Medical Center, Vanderbilt University, Nashville, TN, United States; 4 Department of Neurology and Neurological Sciences, School of Medicine, Stanford University, Stanford, CA, United States; 5 School of Medicine, Stanford University, Stanford, CA, United States; 6 Stanford Health Care, Stanford, CA, United States

**Keywords:** emergency service, referral and consultation, interdisciplinary communication, neurology, ambulatory care, hospital

## Abstract

**Background:**

Given the rising demand for emergency department (ED) services and coupled with the scarcity of specialty care availability, there is an urgency to design a system for appropriate, effective, and timely ED-to-specialty ambulatory referrals. Efficient care transitions are important to patient outcomes and experience and require cross-specialty cooperation, as care transitions affect practices and resources of individuals, departments, and institutions.

**Objective:**

Here, our objective was to (1) describe a collaboration between Stanford’s Emergency Medicine and Neurology and Neurological Sciences departments aimed at designing and implementing an optimized discharge process and transition of care from ED to ambulatory neurology for follow-up care and (2) the resulting intervention from the collaboration.

**Methods:**

We describe the process for barrier identification, tools used to foster partnership and intervention ideation, and the resulting intervention. Our experience and findings are integrated into a 4-component framework for future interdepartmental collaborations: (1) cross-specialty team meetings, (2) preimplementation interviews, (3) a design thinking focus group session, and (4) small group meetings. Qualitative data included observational notes and document review from biweekly cross-specialty and small group meetings, preimplementation interviews, and design-thinking focus groups.

**Results:**

Our process included 10 cross-specialty team meetings with 8 physicians and operational representatives, 18 individual preimplementation interviews, 10 focus group participants, and 9 small group meetings. The process components fostered collaboration and teamwork among the multidisciplinary team; supported early identification of barriers and facilitators, including divergent understanding of project goals; and developed creative ideas that contributed to intervention development. The collaboration resulted in a 4-pronged multimodal intervention. Two elements focused on modifying clinical practice to better triage clinically appropriate ED referrals to ambulatory neurology: (1) optimizing management of conditions in the ED to reduce preventable referrals, and (2) increasing deferral to Primary Care clinicians to direct appropriate specialty follow-up care. Two additional structural elements sought to directly improve appropriate referral timeliness by (3) streamlining insurance authorization processes and (4) increasing neurology appointment availability.

**Conclusions:**

This cross-specialty collaboration resulted in a multimodal intervention that called for both structural and practice changes, which were novel and supported by the results of comparable interventions. Future applications of this framework can validate its utility among different collaborative groups in new settings.

## Introduction

Emergency departments (EDs) are critical interfaces between acute care and the broader health care system, requiring coordination with multiple specialties to provide comprehensive patient care. ED visits among adults have been increasing, with the United States reporting 155 million ED visits in 2022, equating to 47 visits per 100 people [1]. This sustained growth in ED usage, coupled with persistent shortages in specialty care, emphasizes the importance of effective ED-to-specialty referral processes for continuous, comprehensive care [2-4].

Shortages in neurological specialty care are pronounced, a significant threat as characterized by the American Academy of Neurology [5,6]. Nationally, challenges in neurology include longer wait times for appointments compared with other specialty clinics, difficulties filling positions, and establishing care for Medicaid patients [5]. Neurology clinics have competing needs where they must balance referrals from the hospital, ED, and community physicians with their existing case load [7].

Traditionally, health care resource allocation has followed a “more is better” paradigm, assuming that increased availability translates to improved access [8,9]. However, this approach fails to account for real-world limitations such as space and availability of specialty clinicians [8-10]. Given finite resources and growing demand, health care systems must shift toward optimization of resources through strategic, evidence-based interventions that address both clinical practices and structural barriers [11,12].

Increasingly recognized as a key to comprehensive health care, interdisciplinary collaboration is associated with increased patient safety, lower hospitalization rates, and reduced rates of complications and medical errors [13,14]. However, traditional approaches to developing such collaborative interventions are often expert-driven and may not adequately address end user needs or contextual complexities.

Design thinking, a human-centered approach to problem-solving, has emerged as a promising methodology in health care. Evidence suggests that design thinking results in more usable, acceptable, and effective interventions compared to traditional approaches [15]. This approach emphasizes stakeholder engagement, iterative refinement, and empathy-driven design, making it particularly well-suited for complex health care challenges that require coordination across multiple disciplines and care settings.

Using this foundation, we describe the collaboration between the Stanford Department of ED and the Stanford Department of Neurology and Neurological Sciences (hereinafter “Neurology”) that aimed to design and implement an optimized discharge process and transition of care. Here, we describe the resulting intervention and our novel framework that integrates design thinking principles with cross-specialty collaboration.

## Methods

### Setting

#### Institutional Setting

The ED and Neurology departments collaborated on a quality improvement (QI) project from November 2022 to July 2023 at Stanford Medicine, a quaternary academic medical center that serves the Bay Area of Northern California, United States. Within our medical center, both departments are overseen by the same operational service line that provides member departments with strategic operations support and shared infrastructure. The QI initiatives described here were under the Stanford Improvement Capability Development Program [16].

The ED is staffed by 90 physicians (60 clinical full-time equivalents) and 60 trainees (15 per residency class) and conducts 95,000 ED encounters yearly. Neurology provides inpatient and ambulatory services. Ambulatory care includes 9 satellite clinics with 66 physicians, 7 advanced practice providers, 9 nurses, and 60 trainees (residents and fellows) and delivers 48,000 ambulatory encounters yearly. The inpatient service includes 3 consult services (Neurohospitalist, Stroke, and Neurocritical Care) and 4 admitting services (Neurohospitalist, Stroke, Neurocritical Care, and Epilepsy Monitoring Unit). Stanford is a Comprehensive Stroke Center and a Level I Trauma Center.

#### Project Origin

To improve care transitions between emergency services and ambulatory specialty care, the ED first partnered with the Department of Orthopedic Surgery. In June 2022, this partnership implemented a workflow that triaged ED referrals to orthopedic surgery as “routine,” “urgent,” or “immediate,” with specified follow-up timelines; results will be reported elsewhere. Following this collaboration, the ED and Neurology departments partnered on a similar QI initiative, as the ED places the second most referrals to ambulatory Neurology. Per month, the ED sends approximately 72 referrals to Neurology, and approximately 40 are scheduled with an appointment. The most common referrals are for headache or migraine (230 per year) and dizziness or vertigo (58 per year).

The Stanford Medicine Evaluation Sciences Unit (ESU) joined this team to provide enhanced evaluation support. The ESU is a group of clinicians and nonclinicians, doctorate- and master’s-trained faculty, and staff with methodological expertise in evaluation and health services research [16].

Clinical and evaluation members who participated in this collaborative project will be referred to as the cross-specialty team.

### Data Collection

#### Overview

The ESU collected qualitative data throughout the partnership to assess participant perspectives, document collaborative processes, and provide rapid feedback. This included defining and reflecting on goals, intervention, and implementation. Qualitative data included observational notes and document review from biweekly cross-specialty team meetings (Process Component 1), preimplementation interviews [17] (Process Component 2), design-thinking [18] focus group (Process Component 3), and observational notes and document review from ad hoc individual or small group meetings (Process Component 4).

#### Meeting Observations—Cross-Specialty Team Meetings and Small Group Meeting Observations (Process Components 1 and 4)

The cross-specialty team held biweekly meetings on Zoom (Zoom Video Communications) from November 2022 to June 2023. Meetings aimed to develop a shared goal, an intervention, and an implementation approach centered around transitioning patients from ED to ambulatory neurology for follow-up care. Meeting attendees could include physicians, QI consultants, new patient coordinators (NPCs), service line managers, and ESU. Small group meetings focused on gathering in-depth information on department-specific issues; these mostly occurred between Neurology and ESU.

The ESU provided documentation, rapid feedback, and technical assistance during meetings. Two ESU team members (SRH and CB-J) took detailed observational notes to capture discussion and decision-making around goals, intervention, and implementation approaches. Rapid ethnographic principles were followed for observation notes [19]. The ESU collected meeting documents and products, for example, PowerPoints, spreadsheets, PDFs, and screenshots, to capture the development of the intervention.

In addition, the ESU (SRH, CB-J, and JGS) provided rapid, data-informed feedback to the team following methods outlined in the Rapid Implementation Framework (RIF) [20]. Finally, the ESU answered questions and contributed insights related to their expertise, such as primary care (JGS), local QI projects on care coordination (JGS, SMRK, and CB-J), and implementation science (JGS, SMRK, and CB-J) [21].

#### Preimplementation Interviews (Process Component 2)

Semistructured interviews (Table 1) were informed by the premortem approach [17], RIF [20], and the Stanford Lightning Report [22]. Semistructured interview guides (Multimedia Appendix 1) were designed to capture barriers and facilitators to implementation and hypothetical reasons for project failure. The study used a purposive sampling strategy, including the cross-specialty team and key stakeholders identified through snowball sampling. Cross-specialty team members identified additional stakeholders (ie, information technology staff and chief residents) during their interviews. Prospective participants received an email invitation to participate from the ESU. Two ESU team members (SRH and HW) conducted interviews from January to April 2023, recording via Zoom with verbatim transcription (Rev Inc).

**Table 1.** Roles of preimplementation interview participants and team members in a cross-specialty collaboration.

Role	Number of preimplementation interview participants	Number of cross-specialty team members^a^
Physicians	10	5
Operations and technology solutions	5	3
Scheduling	3	0
Total	18	8

a Cross-specialty team denotes participants who attended cross-specialty team meetings (Process Component 1)

#### Design Thinking Focus Group (Process Component 3)

In March 2023, ESU facilitated a 1.5-hour design thinking focus group discussion via Zoom. The session had four goals: (1) better understand potential intervention users, (2) challenge initial assumptions, (3) redefine problems to be user-focused, and (4) create innovative solutions to prototype and test. A total of 10 people from the cross-specialty collaboration team attended, representing Neurology, ED, Primary Care, ESU, Operations, and Quality Consulting. The session included a brief introduction to design thinking and three activities: empathy mapping [23], the worst possible idea [24], and brainstorming [25]. Participants completed an anonymous feedback survey (Multimedia Appendix 2).

### Data Analysis

Rapid analysis methods [20,22] were applied to interview data to synthesize and provide prompt, actionable feedback to the cross-specialty team and capture iterations of the intervention. Two coauthors (SRH and HW) used a priori codes in a matrix analysis [26] in Excel (Microsoft Corp). Early results were presented to the cross-specialty team during a biweekly meeting to validate findings and in small group sessions to generate feedback.

Data from observational notes and document review were used to triangulate findings reported in the preimplementation interviews. Observational notes and interview transcripts were revisited as needed to validate or query findings. For the design thinking focus group, data were analyzed into three broad categories: what worked, what did not work, and ideas for future sessions.

### Ethical Considerations

This project received a nonresearch determination by Stanford University’s institutional review board (IRB #68792). Interview participants gave verbal consent before each recorded interview. Participants were told their privacy and confidentiality would be maintained. Interview participants were not compensated. As this is a report on the early phases of a QI project focused on cross-specialty collaboration, we did not involve patients or the public in the research design, conduct, reporting, or dissemination. However, we sought to include diverse health care employees to understand workflow perspectives. Participants did not receive any form of compensation for participation. The data supporting this study’s findings are available on reasonable request.

## Results

### Novel Framework to Increase Cross-Specialty Collaboration

**Figure 1.**
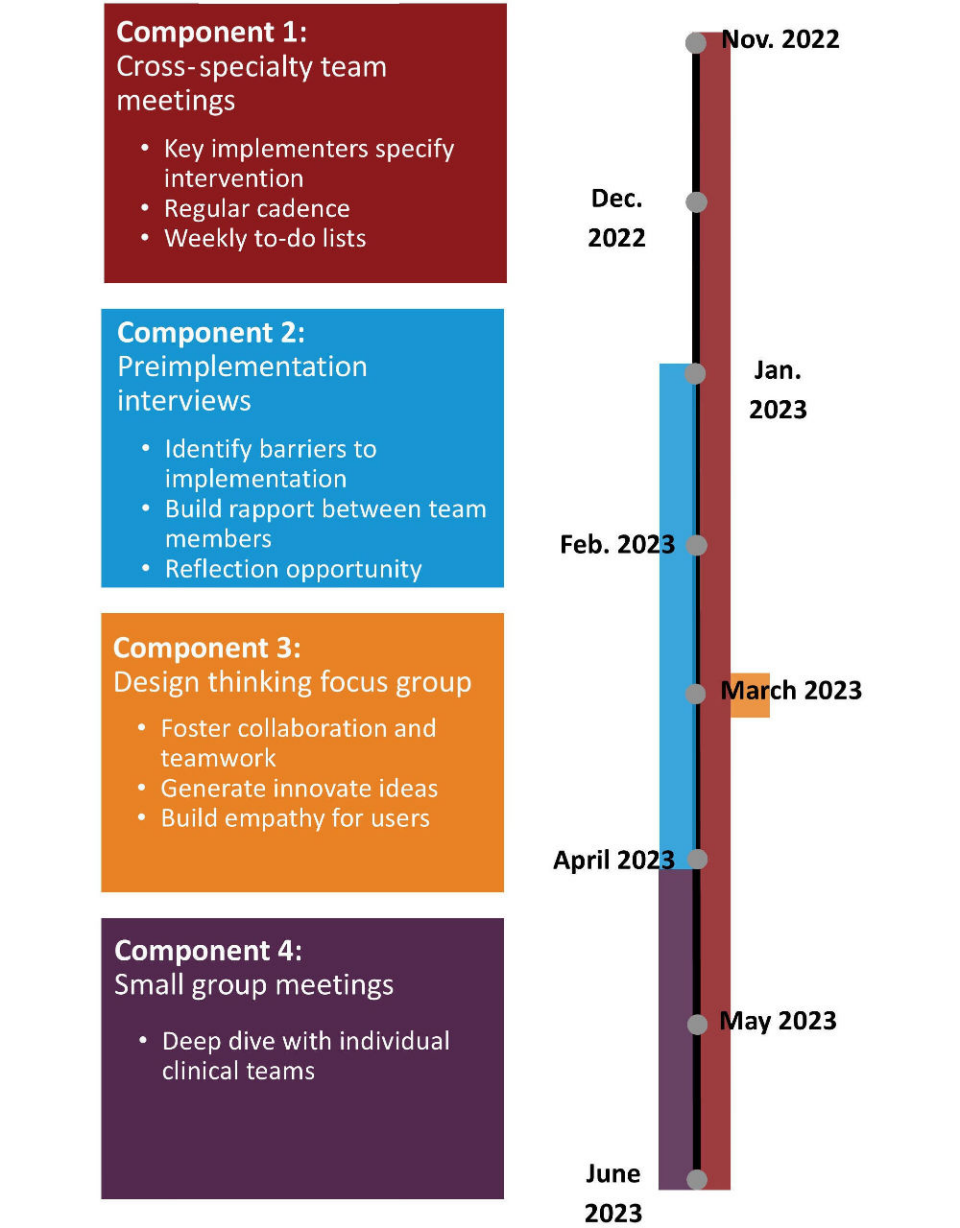
Four-component framework that was used to support cross-specialty collaboration to design and implement an optimized discharge process and transition of care from emergency department to ambulatory Neurology for follow-up care.

This cross-specialty collaboration with enhanced evaluation support identified core activities and milestones for intervention development and implementation. Major process components of our proposed Framework to Increase Cross-Specialty Collaboration (Figure 1) include (1) cross-specialty team meetings, (2) preimplementation interviews, (3) design thinking focus group session, and (4) small group meetings.

Engaging in these process components fostered collaboration and teamwork among the multidisciplinary team, supported early identification of barriers and facilitators, including divergent understanding of project goals and identification of creative ideas that contributed to intervention development.

The process resulted in a multimodal intervention that was informed by qualitative data (as described in Table 2), that addressed diverse needs and perspectives, and aimed to enhance quality through greater attention toward timeliness and appropriateness of ED-to-Neurology referrals. The intervention included two modifications to clinical practice and two structural changes. Element 1, enhancing clinical practices through procedures or treatments, optimized clinical management of conditions in the ED to reduce ED-to-Neurology referrals. For example, increasing the ED’s capacity to perform occipital nerve blocks to eliminate the need for referral. Element 2 embedded a referral considerations document within the electronic medical record, guiding physicians toward primary care or neurology follow-up when appropriate. Element 3 addressed timeliness by streamlining insurance authorization by having financial counselors call patients who could not be seen by Neurology. Element 4 addressed access by adding three Neurology appointment slots per week by the headache specialist and a potential weekend or night clinic.

**Table 2.** The multimodal intervention that resulted from a cross-specialty collaboration that aimed to design and implement an optimized discharge process and transition of care from an ED^a^ to ambulatory neurology for follow-up^b^.

Element of intervention	Description of element	Specific theme or learning from the collaboration framework (process component) that informed the element
Increasing ED clinical practice (practice change)	Investigated procedures or treatments (eg, occipital nerve block) that could be administered in the ED, in lieu of an urgent referral Procedures would not require prior insurance authorization in the ED ED physicians received specific training from Neurology	Creative brainstorming during the design thinking focus group led to the idea for optimizing the management of conditions in the ED through increasing clinical practice Collaborative meeting time used to strategize to implement into practice Conversations to address divergent understanding of project goals led to recognition that timeliness and appropriateness are intertwined, and both needed to be addressed
Use all referral services (practice change)	Focus on headache or migraine and dizziness Increase the percentage of complex cases to neurology Coordinate care with Primary Care for appropriate cases The framework defines appropriate referrals to a primary care clinician (PCP^c^) versus specialty referral to Neurology Short two-sentence commentary embedded into the Epic electronic health record (Epic Systems Corporation) referral order, as a point of order entry education Detailed diagnosis-specific consideration document linked at the point of order entry	The decision to focus attention on two diagnoses (headache and dizziness) emerged after rapid implementation feedback from interviews Primary Care perspective integrated after conversations in cross-specialty collaboration meetings Tensions related to appropriateness versus timeliness led to a need for a referral consideration document Two-sentence commentary and detailed description reviewed during cross-specialty collaboration meetings
Streamline insurance authorization (structural change)	NPCs^d^ spend approximately 1‐4 hours per week calling patients who cannot be scheduled due to insurance Causes frustration for both NPCs and patients New model to change this responsibility from NPCs to finance counselors	Emerged in response to problem statements for the scheduler identified during the design thinking session
Increase access in neurology (structural change)	Stanford headache specialist (3 total) each open 3 urgent slots per patients from the ED with an urgent referral As of July 2023, awaiting approval for a weekend or night clinic and APP^e^ transition clinic	In response to the goal of increasing the timeliness of referrals for urgent cases In response to failure points highlighted in the preimplementation interviews, increasing access occurred as a final step after addressing insurance coverage, and appropriateness of referral

a ED: emergency department.

b Intervention elements were mapped to the components of the collaboration framework that most informed the element.

c PCP: primary care physician.

d NPC: new patient coordinator.

e APP: advanced practice provider.

### Cross-Specialty Team Meetings (Process Component 1)

The team met bimonthly from November 2022 to May 2023 (10 meetings; range 1‐2 h; Figure 1). Team members (5‐15 per meeting) included clinical, scheduling, QI, operations, and ESU. Members of the cross-specialty team attributed meeting success to having regular cadence, a primary ED leader, team-specific action items, and attendance by at least one physician from both ED and Neurology.

The initial meetings were attended by operations, schedulers, and physicians from Neurology and ED. The administrative director for quality and operations for the service line provided oversight and served as liaison between the cross-specialty team and operations. Initial aims included identifying all applicable contributors and establishing a specific, measurable goal for the collaboration. In these meetings, ED discussed prior experience with Orthopedics.

The ESU used the cross-specialty team meetings in the middle and late stages of the collaboration to provide rapid feedback [20] informed by the preimplementation interviews, design thinking focus group, and small group meetings (Process Components 2‐4). Two of the most prominent factors of the RIF reports from February to March 2023 were (1) hypothetical barriers to implementation, that is, limited neurology appointments; and (2) tensions between departments caused by divergent understanding of project goals. To address concerns regarding limited neurology appointments, the project team decided to focus only on the two most common reasons for referral—headaches and dizziness. The team also invited a Primary Care representative to these meetings, recognizing Primary Care’s role in the intervention. From March to May 2023, cross-specialty team meetings (Table 2) focused on developing and launching the intervention to enhance Primary Care usage.

### Component 2: Preimplementation Interviews

#### Overview

Interviews with participants (Table 1) asked about elements necessary for success and potential threats that might thwart success (“premortem” [17] questioning). Interviews identified a need for trust, divergent understanding of project goals, and potential failure points, all of which contributed to goal refinement and intervention development.

#### Need for Trust

Interviewees recognized that the process to refer and coordinate follow-up care post-ED discharge involves many team members and workflows housed in multiple departments; here, this included ED, Neurology, and Primary Care. Proposing to change another team’s processes concerned interviewees and required them to trust that team members were working collaboratively. One participant reflected:

*We are asking another service to change their processes and we can only suggest so much*.

[Participant 10, ED]

#### Divergent Understanding of Project Goals

Interviews revealed that team members had different views on the intended project goal, contributing to an “us and them” (Participant 9, Neurology) dynamic. Interviewed participants described the collaboration’s goal as either to increase the timeliness of Neurology follow-up care or the appropriateness of ED referrals to Neurology.

Although both timeliness and appropriateness are factors influencing patient access to care, approaches differed depending on the driving aim. The ED prioritized timeliness by focusing on different levels of referral priorities (ie, routine, urgent, and immediate) tied to defined timelines similar to their experience with Orthopedic Surgery. In contrast, Neurology prioritized appropriateness, proposing to define the clinical characteristics of patients needing a Neurology referral versus those needing a primary care referral, for example.

Through our collaborative approach, including regular meetings, design thinking sessions, and evaluation team rapid feedback, the participants came to recognize the intertwined nature and necessity to address both timeliness and appropriateness.

#### Potential Failure Points

##### Overview

Preimplementation interviews also asked participants to anonymously name potential failure points, allowing team members to explore sensitive barriers. Potential failure points included the need for widespread education for frontline clinicians, balancing capacity, and insurance coverage.

##### ED Education

Interviewees recognized that any intervention would require widespread ED education. One participant flagged a concern about scale:

*[when] you’re teaching, you can teach one or five people, but this is a whole department.*

[Participant 3, Neurology]

This would be further complicated by resident rotations and the need for continuously available, asynchronous education.

##### Limited Capacity to Increase Access

Interviewees, especially those from Neurology, were concerned that current capacity limited intervention options and that the project itself could exacerbate capacity issues. Interviewees reported that their clinics had substantial wait times for appointments and overburdened schedules as urgent post-ED or hospital follow-ups were being scheduled outside of clinic hours. Balancing capacity issues with other concerns was of clear concern.

*If we expect that patients will follow up in a certain time period, but at the end of the day the capacity of providers and clinics doesn’t match that expectation, then it may fail*.

[Participant 10, ED]

##### Insurance Coverage

Interviewees identified that health insurance coverage may complicate the referral process and patient expectations for follow-up care. Referring a patient to appropriate follow-up care was a top priority to interviewees, but depending on the patients’ insurance, the process of referral was not straightforward.

*Coming up with a plan that’s the same no matter someone’s insurance status is part of our mission ... If I send a referral to someone who is under no medical, legal responsibility to have to follow up with a patient, they might not, not because they disagree with the management plan of the ED*.

[Participant 1, ED]

### Design Thinking Focus Group (Process Component 3)

#### Overview

**Figure 2.**
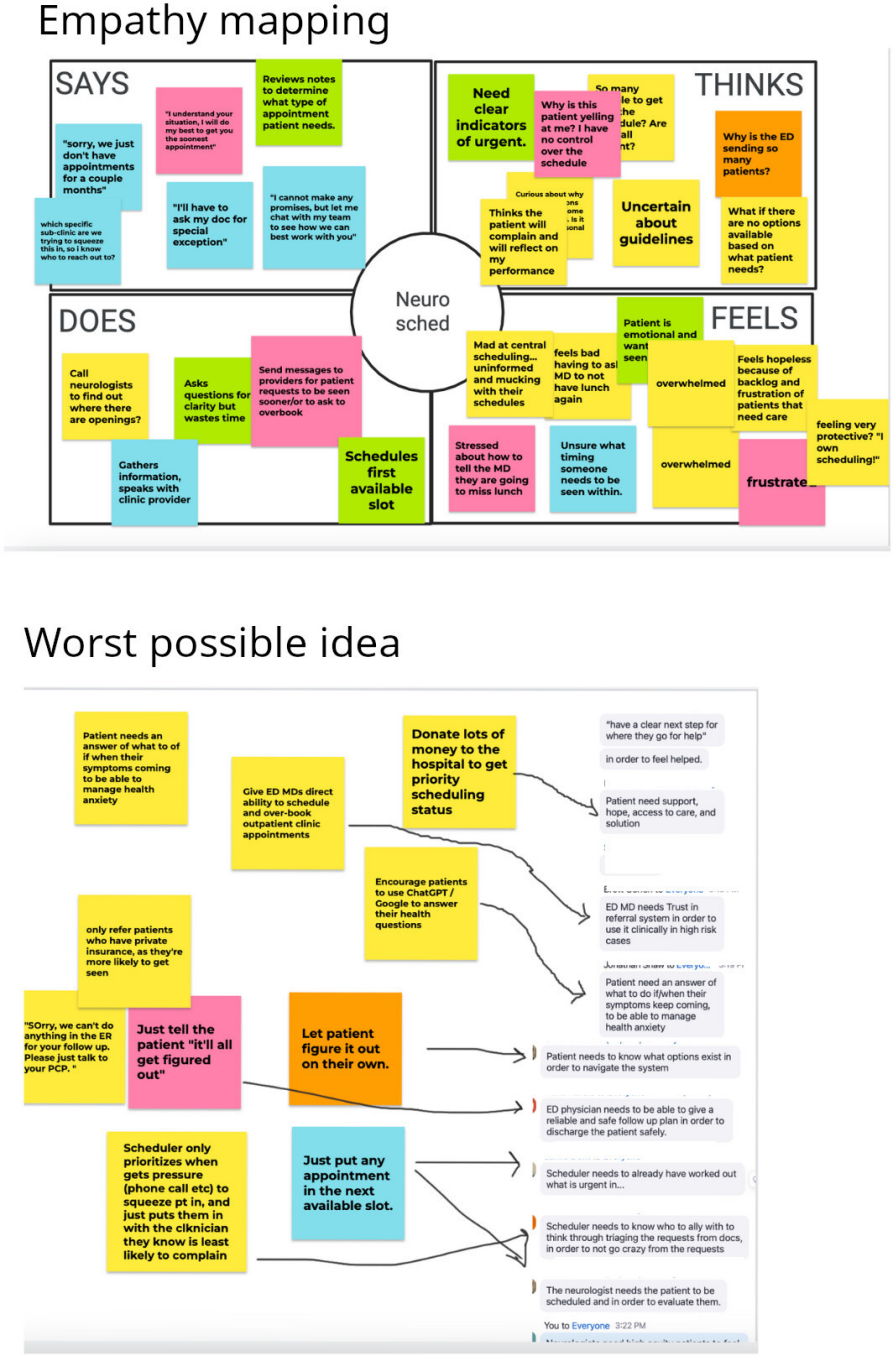
Results of empathy mapping and worst possible ideas from the design thinking focus group that was a component of the framework to support cross-specialty collaboration to optimize the discharge process and transition of care from the emergency department (ED) to ambulatory neurology for follow-up care.

The design thinking focus group session (March 30, 2023), which was based on published best practices [18], engaged team members in 3 activities: empathy mapping, worst possible idea, and brainstorming (Figure 2).

#### Empathy Mapping

Participating team members jointly identified 3 personas (hypothetical end users) affected by the problem and the proposed interventions: ED physician, an ED patient with a migraine who has tried three different medications, and an NPCor scheduler in Neurology. The team was split into two arbitrary groups. Per the empathy mapping [23] protocol, each individual team member was given a few minutes to note what each persona might say, think, do, and feel on their team’s web-based whiteboards (JamBoard; Google Inc). For example, team members noted that an NPC may say [to a patient]: “Sorry, we just don’t have appointments for a couple months,” which might make the NPC think that they need “more clarity on what is urgent.” The NPC might “call a neurologist to try to find a slot,” which makes the NPC feel “overwhelmed and frustrated.”

Teams discussed their whiteboards, summarized findings, and then summarized the goal of each persona as a large group, including (1) patients want to be seen by a clinician and feel better, and (2) Neurology NPCs want to schedule a patient without unnecessary burden and frustration. Team members noted the lack of control and high level of uncertainty for all roles engaged, including patients, physicians, and staff, in the existing referral system.

#### Worst Possible Idea

Small groups created a list of the least effective ideas to address problems identified through “empathy mapping.” One of the “worst possible ideas” was to make clinic staff and clinicians work 18-hour shifts to accommodate all referrals, overburdening clinical staff and creating burnout. This led participants to discuss the importance of not significantly increasing the workload for any staff.

#### Brainstorming

The session ended with a brainstorming [25,27] discussion wherein participants were encouraged to generate all ideas, big or small, without hesitation or consideration of practical limitations. Ideas included (1) all ED patients to go to primary care first to determine need for a Neurology consult, (2) a Neurology “doctor of the day” who is available to see urgent Neurology patients in ambulatory settings, and (3) a Neurology express care model.

#### Design Thinking Feedback Survey

Survey participants (n=8) reported that the design thinking session fostered collaboration and teamwork, generated innovative ideas, and built empathy for users. Participants reported they felt high engagement, were interested in the design thinking process, and were challenged by the activities. A few participants suggested that design thinking exercises would have been helpful earlier in the collaboration because of the emphasis on generating innovative solutions.

Problem statements identified during empathy mapping and ideas generated during brainstorming contributed to the final multimodal intervention, for example, streamlined insurance authorization to address NPC needs.

### Component 4: Small Group Meetings

The ESU met with individual or small groups, usually Neurology, ad hoc to explore emerging work thought to be peripherally related to the primary objective of the cross-specialty team meetings (Figure 1). These small group meetings were used to document intervention development progress in detail and then prioritize what to discuss in the larger implementation meetings. Small group meetings were essential for capturing the two structural changes: (1) streamlined insurance authorization processes and (2) increasing Neurology appointment availability (Table 2).

## Discussion

### Principal Findings

Given rising demand for ED services [28], coupled with a scarcity of specialty care availability [5], the urgency for effective and prompt ED-to-specialty referrals has never been more pronounced. To address this need, we outlined the process used by a cross-specialty collaboration at a single institution and a subsequent multimodal intervention aimed at enhancing quality through appropriateness and timeliness of ED-to-Neurology referrals. The intervention included two elements focused on modifying clinical practice: (1) optimizing management of the most common conditions like headache and dizziness in the ED to reduce preventable referrals, and (2) enhancing the use of Primary Care services to direct specialty follow-up care as needed. Two additional structural elements sought to directly improve referral timeliness by (3) streamlining insurance authorization processes and (4) increasing Neurology appointment availability.

The cross-specialty initiative was enabled through an iterative collaboration process that included regular cross-specialty team meetings, preimplementation interviews, a design thinking focus group session, and small group meetings. Each component provided key insights that shaped the development of the intervention elements and helped build rapport between specialties. Our experience informed the proposed Framework to Increase Cross-Specialty Collaboration for future QI endeavors requiring interdepartmental partnership for future collaboration between ED and specialties.

### Significance of Intervention

Our intervention shares commonalities with other successful initiatives aimed at improving transitions from the ED to ambulatory Neurology for follow-up, while also incorporating its own innovative but broadly applicable elements. For example, the emphasis on streamlining referrals and insurance authorization resembles process improvements made in clinics like UCLA Fast Neuro Clinic [29] and Florida Access Clinic [30]. However, our intervention’s focus on optimizing ED management and leveraging Primary Care expertise before referring to Neurology represents a more nuanced approach to ensuring appropriate referrals.

While some access initiatives have focused narrowly on increasing the number of Neurology clinicians or clinic availability [29-31], our intervention considered clinical appropriateness to promote more judicious referrals, as well as supporting ED clinicians to work at the top of their license. This balancing of timeliness and appropriateness through clinical practice and structural changes is a unique contribution based on currently available literature. There were concerns raised by the Neurology team about educating the entire ED on new workflows, but these activities are relatively straightforward for the ED, given existing processes for QI and education at faculty and resident monthly meetings. It was during these monthly meetings that inclusion and exclusion criteria to Neurology referrals versus Primary Care were identified.

### Value of Collaboration and Design Thinking

The proposed Framework to Increase Cross-Specialty Collaboration fostered integration of diverse insights from ED, Neurology, operations, and Primary Care. Rather than moving forward with an intervention based on team assumptions about referral issues and prior experiences, we gathered input directly from participants through techniques such as preimplementation interviews and design thinking. The Framework allowed the team to identify and address four key hurdles to intervention design: (1) “us versus them” mentality; (2) divergent understanding of project goals (appropriateness vs timeliness); (3) narrowing scope to top Neurology referrals (ie, headache and dizziness); and (4) considering longitudinal health services, like Primary Care, for the patient when appropriate.

Existing evidence on the impact of design thinking in health care demonstrates that it improves intervention usability, acceptability, and outcomes compared to traditional expert-driven methods [15]. Indeed, design thinking is increasingly recognized as a promising human-centered approach for complex problem-solving in health care, moving away from authoritarian dynamics toward cocreative environments [15]. It emphasizes empathy, equitable engagement, and continuous refinement based on end user input [32].

Our use of design thinking in the proposed framework helped elicit openness to nontraditional interventions for improving access. By gathering perspectives on lived experiences of physicians and patients, the intervention resolved underlying problems, instead of creating just symptomatic fixes. This human-centered foundation was key in developing a contextualized, multimodal intervention acceptable to both specialties and aligned with end user needs. Collaborative approaches informed by design-thinking principles have also been shown to result in effective and usable intervention elsewhere [15].

### Limitations

This project has three major limitations related to generalizability, effectiveness, and representation. First, this work represents a single academic institution, which allowed us to leverage existing infrastructure but limited generalizability. Furthermore, the ensuing intervention focused on only the two most common reasons for Neurology referrals, headache and dizziness. Second, the implementation, effectiveness, and sustainability of the intervention remain untested. Third, although we included a range of perspectives, we lack frontline nursing and patient perspectives.

Overall, our process showcases the promise of partnership and design thinking to tailor localized improvements in complex systems like ED to specialty care transitions.

### Conclusions

Interventions to optimize ED to specialty service transitions are vital given the increasing demand for ED services and growing shortage of specialty care availability. Our four-component framework enabled trust, rapport, and early identification of barriers and facilitators to implementation while increasing cross-specialty collaboration and was comprised of: (1) cross-specialty team meetings; (2) preimplementation (“premortem”) interviews; (3) a design thinking focus group; and (4) small group meetings. Development of future access interventions can further refine the Framework to Increase Cross-Specialty Collaboration and look to leverage key elements of our intervention: modifications to clinical practice (eg, including occipital block in ED clinician treatment repertoire) and structural changes (eg, streamlining insurance authorization processes).

Ultimately, this collaboration resulted in a multimodal intervention that called for both structural and practice changes, which were novel and supported by the results of comparable interventions. Future applications of this framework can validate its utility among different collaborative groups in new settings.

## Supplementary material

10.2196/22028 Multimedia Appendix 1 The semistructured guide for preimplementation interviews that were designed to capture barriers and facilitators to implementation and hypothetical reasons for failure of the project to optimize the discharge process and transition of care from ED to ambulatory neurology for follow-up care.

10.2196/22028 Multimedia Appendix 2 An anonymous survey administered to participants at the design thinking focus group. The aim of the survey was to evaluate the experience of the focus group participants.

## Abbreviations

ED: emergency department
ESU: Evaluation Sciences Unit
NPC: new patient coordinator
QI: quality improvement
RIF: Rapid Improvement Framework
