# Supplementary material for: A Novel Approach to Care Redesign Collaboration Between Emergency and Specialty Departments: Qualitative Experience Report
Source: JMIR Form Res. 2025 Aug 26;9:e22028. doi: 10.2196/22028 (PMC12380400; doi:10.2196/22028)
Supplement: Multimedia Appendix 1 [file formative-v9-e22028-s001.pdf]

Topic	Interview question	Secondary prompts
Project Background	Tell me about your involvement in this project? What is your understanding of the origins of this project?	
Problem Understanding	What is the problem from your perspective? What is the problem from a leadership perspective? Institution? Collaborators?	What aspects of this problem can you change? What can you not change?
Pre-Implementation Reflection: Failure	Imagine this is in the future and the project has failed – what would it mean to have failed?	Why did it fail? What are all the ways this could fall apart? Who is responsible for this? How can this fail? What would get in the way?
Pre-Implementation Reflection: Success	Imagine this is in the future and the project has succeeded, what does that mean?	How do you know the project is working?
Collaboration Building	How do you build this collaboration between neurology and ED?	How do you feel communication is going across departments?<br><br>How useful is this communication?
Collaboration Process	What's working now and what is getting in the way?	How are you going to know that the intervention is working?
Optimization Understanding	What does optimization mean to you?	Is there an improved way to use the limited availability you have?
Consensus Building	How do you build consensus between groups?	What mechanisms do you have in place?
Communication Practices	What is your understanding of the current state and process of closed loop communication?	
Follow-up Process from the ED	*What is the current follow up process from the ED? Is it useful in relation to this [project]?	
Health Equity	**How do you think the new triage system would support or undermine health equity?	
Health Insurance	**How do you feel patients' insurance impacts equity in the context of this new triage system?	

*ED specific question
**Added to Interview Protocol after noted as emergent themes from early interviews and workgroup meetings
