# Supplementary material for: A Novel Approach to Care Redesign Collaboration Between Emergency and Specialty Departments: Qualitative Experience Report
Source: JMIR Form Res. 2025 Aug 26;9:e22028. doi: 10.2196/22028 (PMC12380400; doi:10.2196/22028)
Supplement: Multimedia Appendix 2 [file formative-v9-e22028-s002.pdf]

1. There was ample time for discussion and idea building
    a. Strongly disagree
    b. Disagree
    c. Neutral
    d. Agree
    e. Strongly agree
2. I felt engaged and interested during today's design thinking session
    a. Strongly disagree
    b. Disagree
    c. Neutral
    d. Agree
    e. Strongly agree
3. The activities challenged me
    a. Strongly disagree
    b. Disagree
    c. Neutral
    d. Agree
    e. Strongly agree
4. The activities interested me
    a. Strongly disagree
    b. Disagree
    c. Neutral
    d. Agree
    e. Strongly agree
5. The activities were relevant to our work
    a. Strongly disagree
    b. Disagree
    c. Neutral
    d. Agree
    e. Strongly agree
6. If there was another design thinking session, I would want to participate
    a. Strongly disagree
    b. Disagree
    c. Neutral
    d. Agree
    e. Strongly agree
7. Is there something else you would like to add?
